# Influence of land use changes on landscape connectivity for North China leopard (*Panthera pardus japonensis*)

**DOI:** 10.1002/ece3.9429

**Published:** 2022-10-17

**Authors:** Guofu Liang, Jingzhen Liu, Hanbo Niu, Shengyan Ding

**Affiliations:** ^1^ Key Laboratory of Geospatial Technology for Middle and Lower Yellow River Regions Henan University, Ministry of Education Kaifeng China; ^2^ National Demonstration Center for Experimental Environment and Planning Education Henan University Kaifeng China

**Keywords:** circuit theory, land use changes, landscape connectivity, North China leopard, Taihang Mountains

## Abstract

North China leopard (*Panthera pardus japonensis*) is the most widespread subspecies of leopard and one of the rare and endangered species in China. It is currently confined to several isolated natural reserves, and little is known about its habitat network connectivity with land use changes. This study was conducted to assess the impacts of land use changes on landscape connectivity for North China leopard in the Great Taihang Region. Circuit theory‐based connectivity models and least‐cost path analyses were used to delineate pathways suitable for species movement, and evaluate the impacts of land use changes on landscape connectivity. The results revealed that there were 37 least‐cost paths in 1990 and 38 in 2020. The area of forest land increased from 57,142.74 km^2^ to 74,836.64 km^2^, with the percentage increasing from 26.61% to 34.85%. In general, the increase in forest land area promoted the landscape connectivity for North China leopard at broad spatial scales. The improvement of landscape connectivity was not always consistent with the land use changes, and there was a slightly decreasing trend on connectivity in some key movement barrier areas with high intensity of human activities. Improving landscape connectivity at broad spatial scales is as important as protecting the habitats (natural reserves) where the species lives. Our study can serve as an example of exploring the relationships between land use changes and landscape connectivity for species conservation at broad spatial scales with limited movement pattern data. This information is proved to be critical for enhancing landscape connectivity for the conservation concern of North China leopard and planning of natural reserves network.

## Graphical abstract

Evaluating landscape connectivity with land use changes for North China leopard is important. We used Circuit theory to delineate pathways for North China leopard movement with limited movement patterns data. The results showed that improving landscape connectivity at a broad spatial scale is as important as protecting the habitats where the species lived.

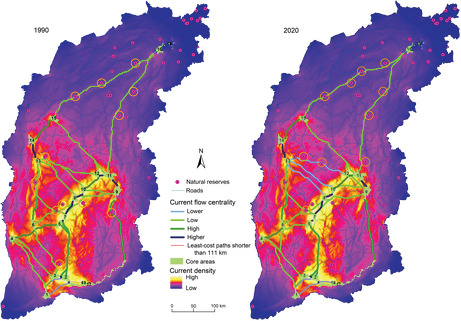

## 1 INTRODUCTION

Land use changes caused by human activities have led to habitat loss and fragmentation at local, regional, landscape, and global scales, which can hinder the migration and dispersal of species at gene, individual, and population levels, further alter the structure and configuration of the landscape, and have become an important factor threatening biodiversity (Ashrafzadeh et al., 2022; Ewers & Didham, 2006; Fischer & Lindenmayer, 2007; Kruess & Tscharntke, 1994). The compounding effects of habitat loss and fragmentation are key drivers of global biodiversity loss (Fahrig, 2003). The loss of natural habitats makes the survival of species very dependent on connections between habitat patches (Baguette et al., 2013; Sahraoui et al., 2017). Landscape connectivity is the degree to which the landscape facilitates or impedes movement among resource patches (Taylor et al., 1993; Tischendorf & Fahring, 2000). Improving landscape connectivity can promote the effectiveness and availability of species habitats (Saura & Pascual‐Hortal, 2007).

Large carnivores are particularly sensitive to landscape fragmentation due to their vast home range and often low population density (Calvignac et al., 2009; Huck et al., 2010; Kaboodvandpour et al., 2021; Mohammadi, Almasieh, Nayeri, et al., 2021). They require large and interconnected habitats with abundant prey for long‐term persistence and viability (Ashrafzadeh et al., 2020; Ripple et al., 2014). Large carnivores are often subject to persecution and conflict with humans, which makes them especially vulnerable to habitat loss and fragmentation (Almasieh et al., 2022; Mohammadi et al., 2022). Conservation of large carnivores requires the preservation of extensive core habitat areas, linkages between them, and mitigation of human‐wildlife conflict (Almasieh et al., 2016; Cushman et al., 2018; Mohammadi, Almasieh, Wan, et al., 2021). High connectivity can facilitate the movement capacity to satisfy ecological requirements and reduce population isolation, and maintaining connectivity is hence recognized as a key factor in the conservation and management of endangered mammal species (Minor & Urban, 2008; Rezaei et al., 2022).

The leopard (*Panthera pardus* Linnaeus, 1758) is widely distributed in the continents of Asia and Africa (Gavashelishvili & Lukarevskiy, 2008). However, because of habitat fragmentation (Selvan et al., 2014) and hunting (Datta et al., 2008), the leopard population has been reduced and is being isolated (Kaboodvandpour et al., 2021). The leopard has been categorized as a vulnerable (VU) species according to the IUCN Red List (Stein et al., 2020). Previous studies have shown that connectivity as well as ground‐level protection and natural forest extent are of profound importance to the distribution and persistence of the leopard population (Farhadinia et al., 2015; Kittle et al., 2018). Conserving leopards requires integrated landscape‐level management to protect corridors and enhance connectivity, especially outside of Protected Areas (Mohammadi et al., 2022). Understanding the impact of fragmentation on leopards and identifying strategies to maintain connectivity are critical to counter the negative impacts of fragmentation (Fattebert et al., 2015; Thatte et al., 2020).

It is important to determine the method for connectivity assessment. The methods may differ from one landscape to another and there is no universal method suitable for all situations. But we can found similar methods in many studies, such as, least‐cost path modeling (Adriaensen et al., 2003; Larue & Nielsen, 2008; Li et al., 2010), graph theory‐based models (Bunn et al., 2000; Devi et al., 2013; Machado et al., 2020; Pascual‐Hortal & Saura, 2006, 2008; Sahraoui et al., 2017; Urban & Keitt, 2001), centrality analyses (Carroll et al., 2012; Estrada & Bodin, 2008), factorial least‐cost path modeling (Cushman et al., 2009; Elliot et al., 2014; Khosravi et al., 2018; Mateo‐Sanchez et al., 2014), resistant kernels (Compton et al., 2007; Cushman & Landguth, 2012; Wasserman et al., 2012) and randomized shortest path algorithms (Long, 2019; Panzacchi et al., 2016; Van Moorter et al., 2021; Vasudev et al., 2021), factorial least‐cost path and cumulative resistant kernel approaches (Ashrafzadeh et al., 2020; Cushman et al., 2013; Kaboodvandpour et al., 2021; Khosravi et al., 2018; McGarigal & Cushman, 2002; Mohammadi, Almasieh, Nayeri, et al., 2021; Shahnaseri et al., 2019), current flow (McRae, 2006), circuit theory (McRae et al., 2008; McRae & Beier, 2007). Circuit theory has already been shown to be useful for predicting movement patterns and probabilities of successful dispersal moving across complex landscapes, generating measures of connectivity or isolation of habitat patches, or protected areas, and identifying important connective elements for conservation planning and designing robust reserve network (An et al., 2021; Dickson et al., 2019; Hanks & Hooten, 2013; Howey, 2011; Koen et al., 2014; Pelletier et al., 2014).

The North China leopard (*Panthera pardus japonensis*, commonly referred to as *Panthera pardus fontanierii* in the Chinese literature) is categorized as class I state key protected wild animals, a top carnivore, and flagship species in the study area and is the most widespread subspecies of leopard and one of the rare and endangered species in China (Laguardia et al., 2017; Liu et al., 2007; Song, 2016; Wang et al., 2001). Recent camera‐trap surveys and other evidence revealed the presence of the North China leopard in Shanxi, Shaanxi, northern Hebei, Ningxia, and northern Henan, and most populations are small, mainly in several isolated nature reserves (Cao et al., 2020; Consolee et al., 2020; Ding & Du, 2020; Gong, 2019; Laguardia et al., 2017; Liu et al., 2007; Song et al., 2014; Vitekere et al., 2020; Zhu et al., 2021). The Great Taihang Region is the dominant distribution area of North China leopard and contains the most densely distributed population of North China leopard in China (Cao et al., 2020). In the past 30 years, with the implementation of a series of forestry projects, such as the National Natural Forest Protection Program, Three‐North Forest Shelterbelt Program, and Grain for Green Project, great changes have taken place in the study area (Li et al., 2017). Yet there is little insight concerning the impacts of land use changes on the landscape connectivity for North China leopard at a large scale.

Landscape connectivity was classified into structural connectivity and functional connectivity. Structural connectivity is equated with habitat contiguity and is measured by analyzing landscape structure, independent of any attributes of the organism(s) of interest, and functional connectivity explicitly considers the behavioral responses of an organism to the various landscape elements (patches and boundaries; Tischendorf & Fahring, 2000). Connectivity can also be divided into three general categories: structural connectivity (derived from physical attributes of the landscape, such as size, shape, and location of habitat patches but does not factor in dispersal ability), potential functional connectivity (based on physical attributes of the landscape with limited data about dispersal ability derived from body size or energy budgets), and actual functional connectivity (based on the observations of individuals moving between focal patches; Calabrese & Fagan, 2004; Fletcher et al., 2016). This study was conducted to assess structural connectivity based on the spatial arrangement of suitable habitat patches (nature reserves) in the landscape, combined with the maximum dispersal ability of North China leopard from body size (potential functional connectivity).

The main objectives of this study were: (1) to detect the land use changes in the Great Taihang Region from 1990 to 2020, (2) to clarify the influences of land use changes on landscape connectivity for North China leopard, and to answer the following research question, (3) how are the changes of the key barrier areas that affect the habitat ecological network connectivity of North China leopard? We hypothesize that land use changes, especially the increase in forest land area, will promote landscape connectivity for North China leopard at a broad spatial scale and the key barrier areas will shrink, and the connectivity of areas with high intensity of human activities will be reduced. This information will be helpful in providing the basis for this unique and rare wildlife species conservation planning in China.

## 2 MATERIALS AND METHODS

### 2.1 Study area

**FIGURE 1:**
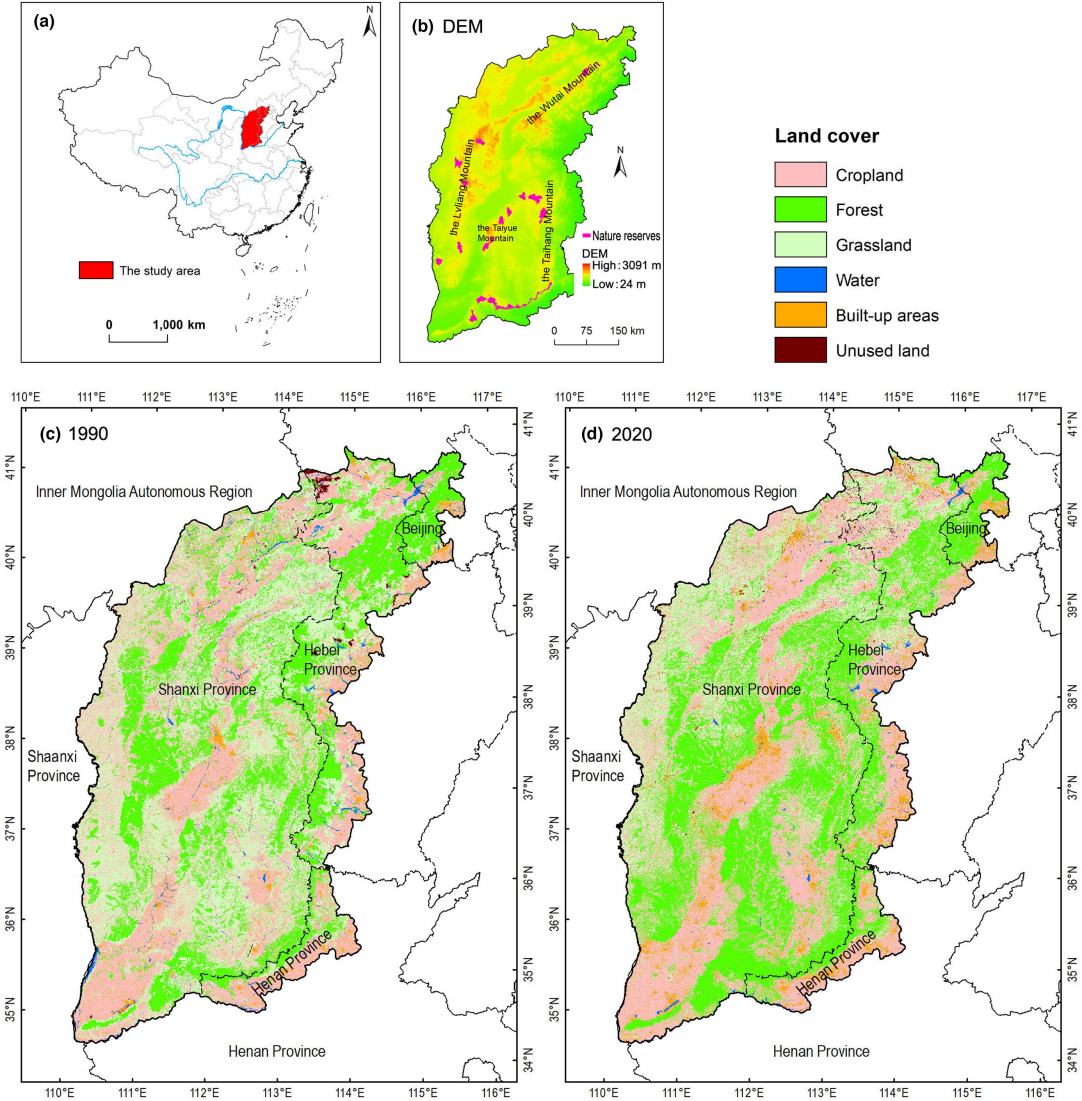
Location of the study area and land cover in 1990 and 2020. (a) location of the study area in China; (b) digital elevation model and nature reserves where the North China leopards are mainly distributed; (c, d) land cover in 1990 and 2020, respectively.

The study area is the Great Taihang Region (34°34′–40°47′N, 110°14′–116°34′E)，with an area of about 214,100 km^2^, located in the north of China and covers the entire territory of Shanxi province, as well as some districts and counties in Beijing, Hebei, and Henan provinces, accounting for about 2.2% of China's terrestrial area (Figure 1) (Cao et al., 2020). In the study area, cropland covers 37% of this area, forest 35%, and Grassland 21%. There are various geomorphic types, including mountains, hills, platforms, and plains. The study area is mainly mountainous area. The eastern part is a massive mountain formed by the Taihang Mountain, and the western part is the Loess Plateau with Lvliang Mountain as the trunk. The altitude ranges between 24 m and 3091 m. The climate in this region is a temperate continental monsoon climate and the four seasons are distinct, with mean annual temperature between 8 and 13°C, and average annual precipitation between 400 and 1000 mm. Rainfall is concentrated from July to September. Deciduous broad‐leaved forest is the most widely distributed plant community in this area. Deciduous broad‐leaved forest and secondary deciduous shrub are mainly distributed in the South, while temperate shrub and semi‐arid grassland are located in the northern part.

The diversity of the geographical environment leads to a good variety of flora and fauna. Among these species, *Panthera pardus* and *Cuon alpinus* were categorized as class I state key protected wild animals in China; *Prionailurus bengalensis*, *Lynx lynx*, *Otocolobus manul*, *Ursus thibetanus*, *Vulpes vulpes*, *Canis lupus*, *Nyctereutes procyonoides*, *Martes flavigula*, *Naemorhedus griseus*, *Macaca mulatta* were classified as class II state key protected wild animals in China; *Panthera pardus*, *Cuon alpinus*, *Otocolobus manul*, *Ursus thibetanus*, *and Naemorhedus griseus* were listed by the IUCN global red list as threatened species (endangered, vulnerable, and near threatened; Table 1; Bu et al., 2021). North China leopard is considered the top predator in their range.

**TABLE 1:** List of the state key protected mammals in China in the study area

Species	Protection status in China	IUCN red list	Red list of China's vertebrate
*Panthera pardus*	I	VU	EN
*Cuon alpinus*	I	EN	EN
*Prionailurus bengalensis*	II	LC	VU
*Lynx lynx*	II	LC	EN
*Otocolobus manul*	II	NT	EN
*Ursus thibetanus*	II	VU	VU
*Vulpes vulpes*	II	LC	NT
*Canis lupus*	II	LC	NT
*Nyctereutes procyonoides*	II	LC	NT
*Martes flavigula*	II	LC	NT
*Naemorhedus griseus*	II	VU	VU
*Macaca mulatta*	II	LC	LC

*Note*: The level of Endangered: EN, endanger; VU, vulnerable; NT, near threatened; LC, least concern.

### 2.2 Circuit theory‐based connectivity models

We used Circuitscape software and cost distance functions to assess landscape connectivity (v4.0, www.circuitscape.org). First, we built nine background layers, including five land use types (cropland, forest land, grassland, water, and built‐up areas), elevation, slope, linear transportation infrastructure, and human population density, which affected species movement (Figure S1; Cao et al., 2020; Petsas et al., 2020; Zhu et al., 2021). Next, these raster maps were converted into a resistance surface based on their values and corresponding weight, reflecting their opposition to species movement.

In each of the five land use type layers, the percentage cover of land use type within every 1 km range was calculated to form a background layer, and different resistance values were assigned according to the percentage cover of land use type in the later stage. For each land use type layer, we assigned the value from 0 (there was no presence of the respective land use type) to 100% (a land use type was full coverage). The elevation and slope data were selected in the background layers to represent topographic features. Data on elevation, slope, and land use were obtained from the National Earth System Science Data Center (http://www.geodata.cn), with a spatial resolution of 30 m. As for the impact of linear transportation infrastructure, we developed a background layer by combining information from expressways, national highways, provincial highways, and railways. The spatial distribution of linear traffic infrastructure datasets was derived from National Catalogue Services for Geographic Information (https://www.webmap.cn; 1:250,000). Each cell was assigned a value, representing the shortest distance from the cell's center to any linear transportation feature, and the spatial resolution was 30 m. The last layer represented human population density, with a spatial resolution of 1 km (Resource and Environment Science and Data Center, https://www.resdc.cn).

We used 1‐km grid to mask the study area, and every single grid (1 km) of the nine background layers was assigned a resistance value ranging from 1 (minimum movement resistance) to 100 (maximum movement resistance) and a weight value ranging from 0 (the layer has no impact on movement decision) to 10 (the layer is very important for movement decision) according to expert opinions (Table S1). Then, we transformed the background layers into resistance layers according to the resistance values and their corresponding weight. Finally, the two resistance surface layers in 1990 and 2020 were created, indicating the impacts of all background layers on North China leopard movement (Figure S2).

### 2.3 Multicollinearity of background layers

To check multicollinearity of the background layers (elevation, slope, distance from roads and railways, and human population density), we established a random sampling system within the study area using the “Random Point” tools in ArcGIS 10.3 (Phompila et al., 2017), producing a total of 5147 random sample points. The attributes of background layers were extracted to each random sample point using the “Extract Multi Values to Points” tools. Then, the spatial correlation between the pair‐wise background layers in 1990 and 2020 was conducted using the Pearson correlation analysis, and the correlation coefficient between selected variables was not excessively high (*r* < |.8|) to avoid multi‐collinearity (Cvitanovic et al., 2016).

### 2.4 Species dispersal distance

There are multiple data on the dispersal distance of leopards, 81.6 km based on the GPS‐satellite Iridium transmitters for *Panthera pardus saxicolor* in natural mountainous areas (Farhadinia et al., 2018), and 352.8 km based on the VHF radio‐collar and GPS/GSM collar for *Panthera pardus* in the Maputaland coastal ecoregion (Fattebert et al., 2013). To the best of our knowledge, it has not been carried out the study on the movement ecology of North China leopard based on GPS collar. According to the allometric growth equations (Santini et al., 2013), we estimated the dispersal capacity of North China leopard with body weight and home range as input data. The adult body weight of North China leopard is about 52.40 kg, and the home range is about 18.78 km^2^ (Jones et al., 2009). The maximum dispersal distance of North China leopard is calculated as *d* = 13.11 × *BS* ^0.34^ × *HR* ^0.27^, *BS* is body weight (kg), *HR* is home range (km^2^), and dispersal distance *d* is in km (about 111 km). In this study, considering the topographic characteristics of the study area, including mountains, hills, platforms, and plains, we lastly determined the species dispersal capacity is 111 km.

### 2.5 Quantifying connectivity properties

We identified landscape connectivity using circuit theory and least‐cost path analyses implemented via Linkage Mapper v.2.0.0. in ArcGIS 10.3 (McRae & Kavanagh, 2011). The core area (18 nature reserves) polygons and resistance raster were used to perform cost‐weighted distance calculations from each nature reserves. The Centrality tools from the Linkage Mapper toolbox were used to calculate current flow centrality across the networks, which measures how important a link or core area is for keeping the overall network connected. We used the Barrier Mapper tool from Linkage Mapper to detect important barrier areas of connectivity restoration (McRae, 2012).

## 3 RESULTS

### 3.1 Land use change status

Table 2 sets out the descriptive statistics of land use type areas and changes from 1990 to 2020. The dominant land use types in the study area are cropland, forest land, and grassland, with the areas of 79,346.29, 74,836.64, and 45,670.23 km^2^ in 2020, accounting for 36.95%, 34.85%, and 21.27% of the study area, respectively. The area of forest land increased from 57,142.74 to 74,836.64 km^2^ at a rate of 655.33 km^2^/a, with the percentage increased from 26.61% to 34.85%. The area of grassland decreased from 69,226.26 to 45,670.23 km^2^ at a rate of 872.45 km^2^/a, with the percentage decreased from 32.23% to 21.27%. Under the influence of human activities, the expansion of built‐up areas was evident with the percentage increased from 3.07% to 5.87%, and the area increased from 6596.23 to 12,605.26 km^2^ at a rate of 222.56 km^2^/a.

**TABLE 2:** Descriptive statistics of land use type areas and changes from 1990 to 2020

Types of land use	Area in 1990 (km^2^)	Area in 2020 (km^2^)	Proportion in 1990	Proportion in 2020	Proportion change from 1990 to 2020
Cropland	78,170.3	79,346.29	36.40%	36.95%	0.55%
Forest land	57,142.74	74,836.64	26.61%	34.85%	8.24%
Grassland	69,226.26	45,670.23	32.23%	21.27%	−10.97%
Water	2830.98	1188.85	1.32%	0.55%	−0.76%
Built‐up areas	6596.23	12,605.26	3.07%	5.87%	2.80%
Unused land	789.80	1109.04	0.37%	0.52%	0.15%
Total	214,756.31	214,756.31	100.00%	100.00%	

### 3.2 Spatial distribution of the cumulative current map and least‐cost paths

**FIGURE 2:**
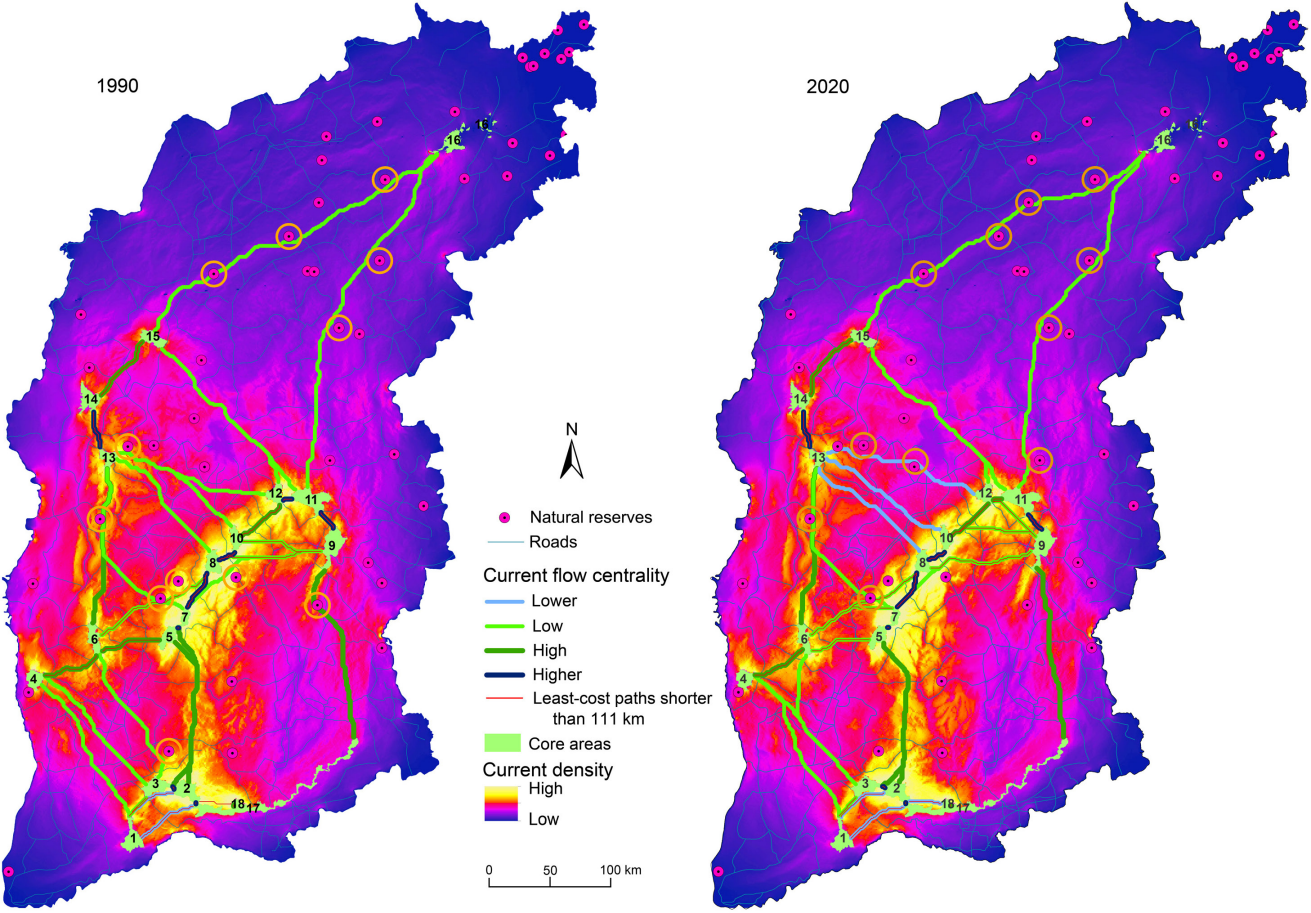
Cumulative current map, least‐cost paths, and current flow centrality results for North China leopard. Core areas were the 18 nature reserves used to model landscape connectivity. The yellow circle indicated that the distance between the center of the nature reserve and the least‐cost path was <10 km.

Multi‐collinearity analysis indicated that the spatial correlation between the pair‐wise background layers (elevation, slope, distance from roads and railways, and human population density) did not exceed 0.8 (Figure S3), and all background layers were preserved. Figure 2 shows the cumulative current map, least‐cost paths, and current flow centrality results for North China leopard. Two main corridors were identified by the cumulative current map. The larger corridor was recognized in the middle of the southern part of the study area, spanning the Taiyue Mountain and some parts of the Taihang Mountain. Another large corridor was detected in the western part of the study area, mainly crossing along the Lvliang Mountains. In 1990, there were 37 least‐cost paths, of which 20 were less than 111 km. Nevertheless, there were 38 least‐cost paths in 2020, with 22 paths less than 111 km. According to the method of natural breakpoints, the current flow centrality is divided into four levels, namely, higher, high, low, and lower. In 1990, the least‐cost paths with higher centrality were among the nature reserve of 3–2‐17, 13–14, 5–7–8‐10, and 9–11‐12, while in 2020, there were no changes except that the link between nature reserves 11 and 12 is no longer in a higher rank. The number of nature reserves (within the yellow circles) with centers less than 10 kilometers away from the least‐cost path was 12 in 1990 and 11 in 2020.

### 3.3 Change of key barriers

**FIGURE 3:**
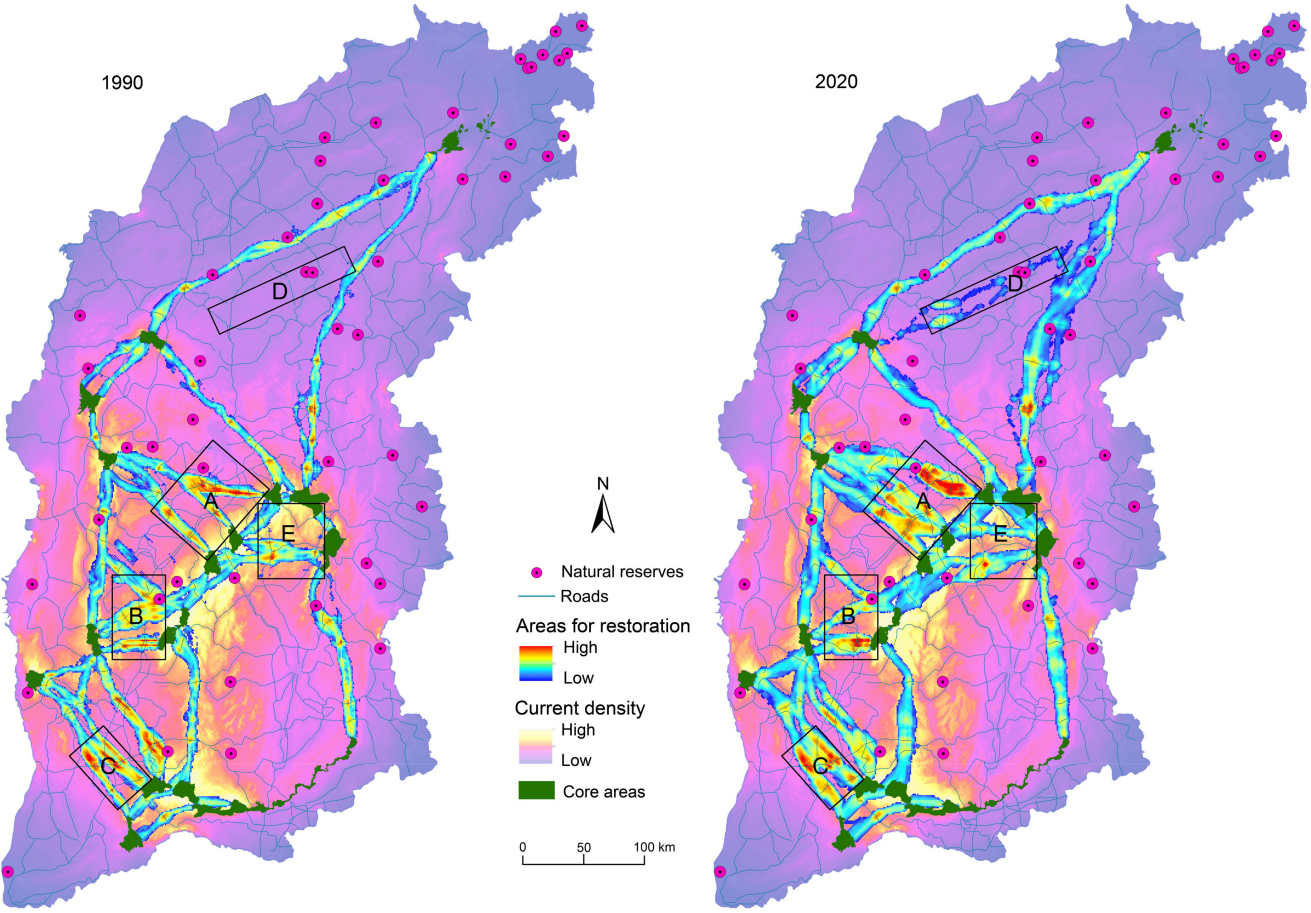
Key barrier areas marked with (a, b, and c), with the greatest values of connectivity restoration potential. In areas (d and e), new connectivity corridors appeared.

The Barrier Mapper analysis identified three key barrier areas (a, b, and c) for restoring connectivity for North China leopard across the whole landscape (Figure 3). As a result of land use changes, within barrier areas a, b, and c, the key barriers to migration corridors did not show a significant downward trend. By contrast, in areas D and E, new connectivity corridors appeared, which were more conducive to improve the connectivity among core areas and promote species movement.

**FIGURE 4:**
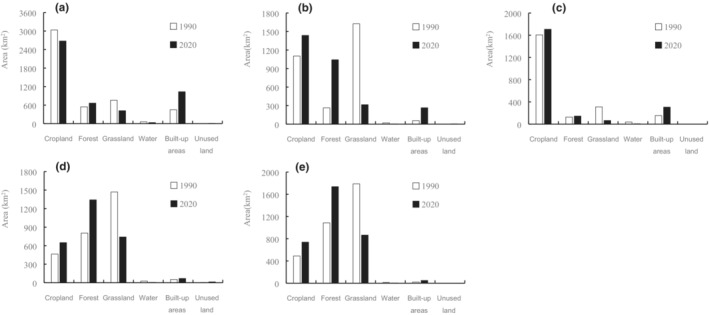
Land use changes within areas (a, b, c, d, and e) from 1990 to 2020.

In barrier areas A and C (Figure 4A,C, Table S2), the land use type was characterized by cropland. In 2020, the proportion of cropland was 55.28% and 76.46%, respectively, and the proportion of forest land was 13.75% and 6.50%, respectively. The proportion of grassland was even lower, 8.67% and 2.95%, respectively, in 2020. From 1990 to 2020, the area of built‐up areas increased by 130.68% and 100.17%, respectively. In barrier area B (Figure 4B, Table S2), in 2020, the proportion of built‐up areas increased by 371.39%, the cropland increased by 30.32%, and the forest land increased by 296.52%, reaching 33.97%. In areas D and E (Figure 4D,E, Table S2), the terrain is mainly mountainous, and forest land and grassland are the main land use types. In 2020, the proportion of forest land was 47.57% and 51.12%, respectively, and the proportion of grassland was 26.27% and 25.48%, respectively. From 1990 to 2020, the area of forest land increased by 66.88% and 60.04%, respectively.

## 4 DISCUSSION

The results achieved in our work showed that land use changes and human activities have most important impacts on landscape connectivity, especially the changes in the migration corridors and key barrier areas. In order to protect North China leopard, we should not only protect the habitats where the species lived but also improve landscape connectivity at broad spatial scales. In this study, we aggregated information from expert opinions and various factors that potentially affect the movement of North China leopard, in an effort to identify the migration corridors and key barrier areas. This could be used as a systematic approach for conservation planning for North China leopard.

### 4.1 Methodological considerations

There are many methods to evaluate landscape connectivity. The factorial least‐cost path and cumulative resistant kernel approaches are also particularly useful when employed in combination to accurately identify core habitats, fracture zones, and corridors across a broad landscape (Cushman et al., 2013; McGarigal & Cushman, 2002). In general, species distribution modeling is applied to predict habitat suitability map, and then, species occurrence data and the resistance layers are used to identify core habitats and corridors using the resistant kernel and factorial least‐cost path methods (Ashrafzadeh et al., 2020; Kaboodvandpour et al., 2021; Mohammadi, Almasieh, Nayeri, et al., 2021; Shahnaseri et al., 2019). In this study, due to the lack of exact species occurrence data, we combined the nature reserves where species lived (as core areas) and the constructed resistance layers to identify migration corridors using the circuit theory‐based connectivity model.

When applying the circuit theory‐based connectivity model to analyze landscape connectivity, it is necessary to determine the landscape resistance surface and species dispersal distance, which is difficult to accurately obtain, especially in the absence of species characteristics data at broad spatial scales (Petsas et al., 2020). In our study, we evaluated the main factors affecting the migration of North China leopard by aggregating information from the literatures and expert opinion. Expert knowledge could not replace the information obtained from actual movement, but it could be valuable (Sahraoui et al., 2017). The accuracy of the data needs to be further verified by field experiments.

### 4.2 Core area and connectivity

There are usually two methods in the selection of the core area. One is to identify discrete wilderness patches from the Boolean wilderness map, and the other is to select areas with the highest wilderness quality index from the wilderness continuum map (Cao et al., 2019). In the study area, the leopard movement data are not available at this stage, so we choose the 18 nature reserves where the north China leopard lived as core areas to assess landscape connectivity. From 1990 to 2020, the location of the nature reserve has not changed, which is one of the reasons why the landscape connectivity has not changed significantly at broad spatial scales. Further research is needed to consider the distribution of more suitable habitats out of nature reserves and their impact on landscape connectivity. However, the careful analysis also shows that nature reserves with centers <10 km away from the least‐cost path are important stepping stone habitats. In the northern part of the study area, especially the link between nature reserves 11–16 and 15–16, strengthening the management of these nature reserves and barrier areas will facilitate the migration and protection of North China leopard.

### 4.3 Contribution of land use changes

Within the barrier areas A and C, lower forest land area and higher cropland area, combining the increase in built‐up area, barriers to migration corridors showed a slight increase in trend. This supports our hypothesis that connectivity of regions with high intensity of human activities will be reduced and concurs with a previous study that vegetation, including grass, shrub, forest, and vegetation density, is a key habitat variable for predicting the occurrence of leopards (Farhadinia et al., 2015; Kaboodvandpour et al., 2021), and leopards occurrence is driven both by vegetation and forest proximity (as a proxy for security from human persecution; Kittle et al., 2018; Mohammadi et al., 2022; Penjor et al., 2018). Cropland may be an important habitat variable indirectly (Farhadinia et al., 2015; Hosseini et al., 2019). Sometimes, leopards may go to villages and croplands to chase livestock, leading to human‐leopard conflict (Parchizadeh & Adibi, 2019). Leopards have a broader habitat niche (Kaszta et al., 2020; Rather et al., 2020; Stein et al., 2020), but relatively high vulnerability to human‐wildlife conflict (Rostro‐Garcia et al., 2016). Distance from agricultural areas is an important variable in habitat suitability (Barashkova et al., 2017; Erfanian et al., 2013).

Within the barrier areas B, barriers to migration corridors tended to slow down. Forest and forest‐shrub land mosaic are important cover types for leopards distribution (Khosravi et al., 2019; Macdonald et al., 2019). The availability of food is an important factor in the choice of habitat for large carnivores, having abundant prey plays a vital role in choosing the habitat and spatial distribution (Aryal et al., 2014). Prey presence and distance to villages were further identified as the major drivers of Persian leopard habitat suitability (Ashrafzadeh et al., 2019). Leopards feed on smaller animals in areas close to villages, while they farther away will also prey on larger animals based on the diet analysis (Henschel et al., 2011), and strongly avoid areas that may encounter humans (Strampelli et al., 2018). Relevant to our study, it is found that common North China leopard prey are mainly wild boar (*Sus scrofa*), roe deer (*Capreolus capreolus*), hare, and pheasant in Tieqiaoshan Provincial Nature Reserve, and the North China leopard prefers to prey on wild boar (Zhu et al., 2021). Due to the large area of Chinese pine forest, the relative abundance index of wild boar was found to be higher than that of roe deer in Tieqiaoshan Provincial Nature Reserve (Zhu et al., 2021). This is why the population density of North China leopards gradually increases after moving away from the village, increases with the distribution of wild boars, and decreases with the distribution of roe deer (Zhu et al., 2021). The larger the area of woody savanna, the higher the population density of North China leopards, which may be due to the higher distribution of the main prey of North China leopards in Tieqiaoshan Provincial Nature Reserve (Zhu et al., 2021). Consistent with our hypothesis, in barrier area B, with the increase in forest land and cropland area, barriers to migration corridors tended to slow down.

In areas D and E, the new migration corridors appeared in 2020, which were more conducive to improve the connectivity between core areas and promote species migration. Forest coverage will affect the habitat utilization and spatial distribution of leopards (Simcharoen et al., 2008). With the increase in forest land area, the patch area of habitat outside natural reserves is also increasing. As the area of suitable habitat increases, new connecting corridors will be created.

### 4.4 The implications for the conservation

Habitat fragmentation is a process in which a large natural habitat is converted into several smaller and spatially separated habitat patches, and this process has significant adverse effects on wildlife populations (Kaboodvandpour et al., 2021). Large carnivores such as North China leopard need vast and highly connected natural habitats to meet their different biological requirements (Zhu et al., 2021). Therefore, highly connected habitat patches are necessary to keep North China leopard alive, and protecting them is an urgent priority (Cao et al., 2020). This study provides important implications for conservation. It can serve as a baseline assessment of landscape connectivity for North China leopard. In this study, detecting key barrier areas to connectivity could be an appropriate approach for North China leopard protection. Conservation actions may be most effective if we focus on the protection of the core areas and key barrier areas among them, with priority given to them to improve the connectivity and increase the likelihood of species movements (Khosravi et al., 2018; Saura et al., 2017).

To maximize the viability of the North China leopard population, we suggest several conservation efforts: (1) maintaining healthy North China leopard population, especially in protected areas, to guarantee the long‐term survival of North China leopards; (2) mitigating human‐leopard conflict, mitigation measures can be taken with local participants to promote the coexistence of human and North China leopard; (3) Strengthening the protection of stepping stone habitats, especially, the nature reserves with centers <10 km away from the least‐cost path; (4) adding new core areas to the protected areas network strategically considering along the routes of the least‐cost path (especially the length of least‐cost path > 111 km) within or near the key barrier areas; and (5) enhancing potential corridors for the North China leopard to facilitate dispersal of individuals between core areas (for example, in area D).

## 5 CONCLUSION

In this paper, we used a circuit theory‐based approach to explore the relationships between land use changes and landscape connectivity for North China leopard. The results confirmed that the increase in forest land area will promote the landscape connectivity for North China leopard at broad spatial scales, and connectivity of regions with high intensity of human activities will be reduced. Our study provides an effective approach for assessing the impacts of land use changes on landscape connectivity for North China leopard at broad spatial scales, specifically, when information on species movement patterns is scarce. Therefore, the results could guide conservation actions and contribute to government decision‐making, so as to enhance landscape connectivity for conservation concern of North China leopard and planning of natural reserves network.

## AUTHOR CONTRIBUTIONS

**Guofu Liang:** Conceptualization (equal); methodology (equal); resources (lead); software (equal); supervision (lead); validation (equal); writing – original draft (lead); writing – review and editing (lead). **Jingzhen Liu:** Conceptualization (equal); methodology (equal); software (lead); validation (equal); writing – original draft (supporting); writing – review and editing (supporting). **Hanbo Niu:** Conceptualization (equal); methodology (equal); software (supporting); validation (equal); writing – original draft (supporting); writing – review and editing (supporting). **Shengyan Ding:** Conceptualization (equal); methodology (equal); supervision (supporting).

## FUNDING INFORMATION

This study was financially supported by the Natural Science Foundation of Henan Province (grant number 202300410095), and the National Demonstration Center for Experimental Environment and Planning Education (Henan University) Funding Project (grant number 2020HGSYJX002).

## CONFLICT OF INTEREST

The authors declare no conflict of interest.

## Supporting information


Appendix S1

## Data Availability

Data used for the analysis are uploaded in a Dryad repository (https://doi.org/10.5061/dryad.msbcc2g23).
