# Supplementary material for: Influence of land use changes on landscape connectivity for North China leopard (Panthera pardus japonensis)
Source: Ecol Evol. 2022 Oct 17;12(10):e9429. doi: 10.1002/ece3.9429 (PMC9596324; doi:10.1002/ece3.9429)
Supplement: Supplementary file 1 — Appendix S1 [file ECE3-12-e9429-s001.docx]

Appendix


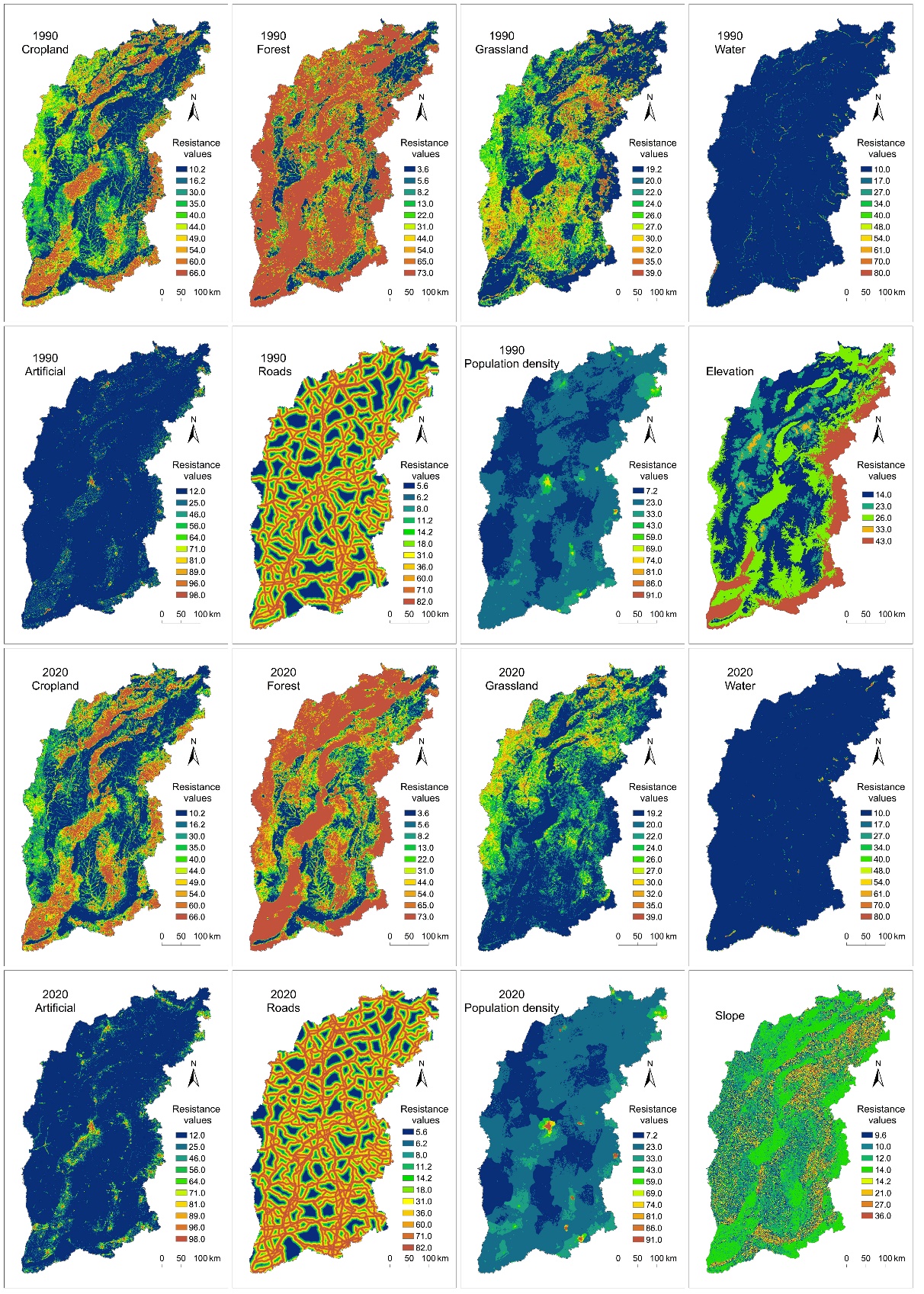


**Figure S1.** Background layers, a total of nine layers, namely, cropland, forest, grassland, water, artificial, distance from roads and railways, human population density, elevation, and slope, used to define landscape properties. Elevation and slope are the same in 1990 and 2020.

**Table S1.** Resistance values and layer importance weights derived from expert opinion to produce resistance surface. Resistance values ranging from 1 (minimum movement resistance) to 100 (maximum movement resistance). Weight values ranging from 0 (the layer has no impact on movement decision) to 10 (the layer is very important for movement decision) according to expert opinions.

| **Background layers** | **Mean resistance values (SD)** | **Importance weights** |
| --- | --- | --- |
| Forest Cover (%) |  | 2.6 |
| [0-10) | 73.0 (17.8) |  |
| [10-20) | 65.0 (19.0) |  |
| [20-30) | 54.0 (19.6) |  |
| [30-40) | 44.0 (19.6) |  |
| [40-50) | 31.0 (14.3) |  |
| [50-60) | 22.0 (9.8) |  |
| [60-70) | 13.0 (6.0) |  |
| [70-80) | 8.2 (2.2) |  |
| [80-90) | 5.6 (2.3) |  |
| [90-100] | 3.6 (3.6) |  |
| Cropland Cover (%) |  | 5.8 |
| [0-10) | 10.2 (10.3) |  |
| [10-20) | 16.2 (17.0) |  |
| [20-30) | 30.0 (31.0) |  |
| [30-40) | 35.0 (29.2) |  |
| [40-50) | 40.0 (27.6) |  |
| [50-60) | 44.0 (25.8) |  |
| [60-70) | 49.0 (22.2) |  |
| [70-80) | 54.0 (19.6) |  |
| [80-90) | 60.0 (15.5) |  |
| [90-100] | 66.0 (12.0) |  |
| Grassland Cover (%) |  | 4.0 |
| [0-10) | 19.2 (18.3) |  |
| [10-20) | 20.0 (15.2) |  |
| [20-30) | 22.0 (11.2) |  |
| [30-40) | 24.0 (8.6) |  |
| [40-50) | 26.0 (8.6) |  |
| [50-60) | 27.0 (9.8) |  |
| [60-70) | 30.0 (14.1) |  |
| [70-80) | 32.0 (18.1) |  |
| [80-90) | 35.0 (23.7) |  |
| [90-100] | 39.0 (30.7) |  |
| Water Bodies Cover (%) |  | 5.6 |
| [0-10) | 10.0 (10.0) |  |
| [10-20) | 17.0 (16.6) |  |
| [20-30) | 27.0 (31.7) |  |
| [30-40) | 34.0 (29.2) |  |
| [40-50) | 40.0 (27.7) |  |
| [50-60) | 48.0 (25.4) |  |
| [60-70) | 54.0 (24.2) |  |
| [70-80) | 61.0 (21.5) |  |
| [80-90) | 70.0 (17.0) |  |
| [90-100] | 80.0 (17.0) |  |

**Table S1**. continued

| **Background layers** | **Mean resistance values (SD)** | **Importance weights** |
| --- | --- | --- |
| Artificial Cover (%) |  | 9.2 |
| [0-10) | 12.0 (4.0) |  |
| [10-20) | 25.0 (12.6) |  |
| [20-30) | 46.0 (27.1) |  |
| [30-40) | 56.0 (22.2) |  |
| [40-50) | 64.0 (18.3) |  |
| [50-60) | 71.0 (14.6) |  |
| [60-70) | 81.0 (9.7) |  |
| [70-80) | 89.0 (6.6) |  |
| [80-90) | 96.0 (2.0) |  |
| [90-100] | 98.0 (1.7) |  |
| Elevation (m) |  | 3.6 |
| [0-500) | 43.0 (18.9) |  |
| [500-1000) | 26.0 (10.2) |  |
| [1000-1500) | 14.0 (9.7) |  |
| [1500-2000) | 23.0 (24.0) |  |
| [2000-∞) | 33.0 (21.4) |  |
| Slope (degrees) |  | 4.0 |
| [0-5) | 14.0 (5.8) |  |
| [5-10) | 12.0 (5.1) |  |
| [10-15) | 10.0 (5.5) |  |
| [15-20) | 9.6 (5.5) |  |
| [20-25) | 14.2 (13.7) |  |
| [25-30) | 21.0 (15.9) |  |
| [30-35) | 27.0 (19.1) |  |
| [35-∞) | 36.0 (26.2) |  |
| Distance from Roads and Railways (km) |  | 4.4 |
| [0-1) | 82.0 (4.0) |  |
| [1-2) | 71.0 (4.9) |  |
| [2-3) | 60.0 (6.3) |  |
| [3-4) | 36.0 (13.2) |  |
| [4-5) | 31.0 (13.6) |  |
| [5-6) | 18.0 (6.8) |  |
| [6-7) | 14.2 (5.6) |  |
| [7-8) | 11.2 (6.3) |  |
| [8-9) | 8.0 (6.4) |  |
| [9-10) | 6.2 (7.0) |  |
| [10-∞) | 5.6 (7.3) |  |
| Human Population Density (people/km^2^) |  | 8.4 |
| [0-100) | 7.2 (6.6) |  |
| [100-500) | 23.0 (14.7) |  |
| [500-1000) | 33.0 (14.0) |  |
| [1000-1500) | 43.0 (15.4) |  |
| [1500-2000) | 59.0 (10.2) |  |
| [2000-2500) | 69.0 (10.7) |  |
| [2500-3000) | 74.0 (8.0) |  |
| [3000-3500) | 81.0 (3.7) |  |
| [3500-4000) | 86.0 (3.7) |  |
| [4000-∞) | 91.0 (5.8) |  |


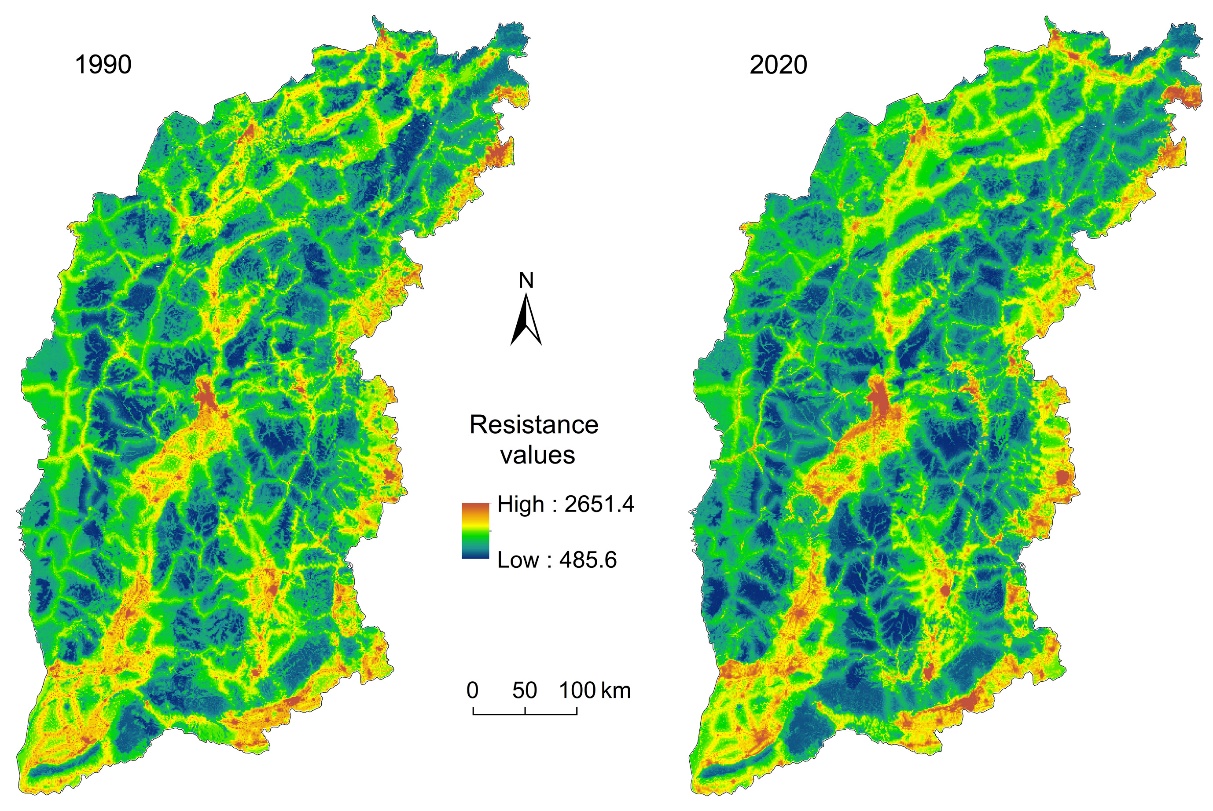


**Figure S2.** Resistance surface layers in 1990 and 2020, indicating the impacts of all background layers on North China leopard movement.


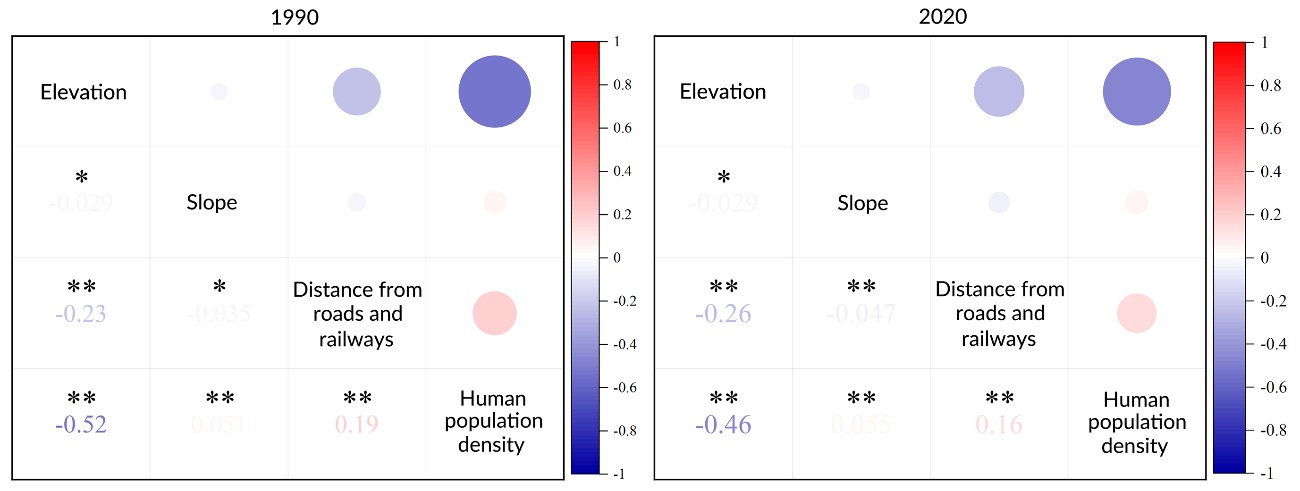


**Figure S3**. The Pearson correlations between pairs of selected variables (elevation, slope, distance from roads and railways and human population density) in 1990 and 2020.

**Table S2.** Descriptive statistics of land use type areas within key barrier areas A, B, C and area D, E in 1990 and 2020. (km^2^)

|  | **Cropland** | **Forest** | **Grassland** | **Water** | **Built-up area** | **Unused land** |
| --- | --- | --- | --- | --- | --- | --- |
| Area A |  |  |  |  |  |  |
| 1990 | 3033.80 | 544.24 | 757.70 | 58.62 | 448.51 | 0.83 |
| 2020 | 2677.36 | 665.79 | 420.18 | 34.77 | 1034.62 | 10.99 |
| Area B |  |  |  |  |  |  |
| 1990 | 1102.91 | 262.95 | 1625.75 | 20.39 | 56.26 | 1.21 |
| 2020 | 1437.34 | 1042.63 | 315.23 | 4.22 | 265.22 | 4.82 |
| Area C |  |  |  |  |  |  |
| 1990 | 1604.98 | 126.95 | 309.98 | 37.22 | 152.88 | 0.95 |
| 2020 | 1707.25 | 145.17 | 65.87 | 6.53 | 306.02 | 2.11 |
| Area D |  |  |  |  |  |  |
| 1990 | 464.11 | 804.32 | 1469.38 | 27.06 | 52.47 | 4.05 |
| 2020 | 649.70 | 1342.25 | 741.20 | 5.63 | 69.47 | 13.14 |
| Area E |  |  |  |  |  |  |
| 1990 | 489.61 | 1085.74 | 1787.29 | 12.70 | 20.26 | 3.17 |
| 2020 | 739.26 | 1737.59 | 865.91 | 5.07 | 49.25 | 1.68 |
